# Dengue: Uncommon Neurological Presentations of a Common Tropical Illness

**DOI:** 10.5005/jp-journals-10071-23179

**Published:** 2019-06

**Authors:** Harpreet Singh, Ashok Kumar Pannu, Ashish Bhalla, Vikas Suri, Savita Kumari

**Affiliations:** 1-5 Department of Internal Medicine, PGIMER, Chandigarh, India

## Abstract

Dengue is a common arthropod-borne flavivirus and commonly manifests as fever, bleeding diathesis, and capillary leak syndrome. Neurological manifestations are uncommon except for encephalopathy. We have recently had the opportunity of observing two patients with rare neurological complications of dengue, transverse myelitis, and Guillain-Barré syndrome. Both the cases had good neurological recovery with steroid and intravenous immunoglobulin, respectively.

**How to cite this article:** Singh H, Pannu AK, Bhalla A, Suri V, Kumari S. Dengue: Uncommon Neurological Presentations of a Common Tropical Illness. Indian J Crit Care Med 2019;23(6):274–275.

## CASE 1

**Fig. 1:**
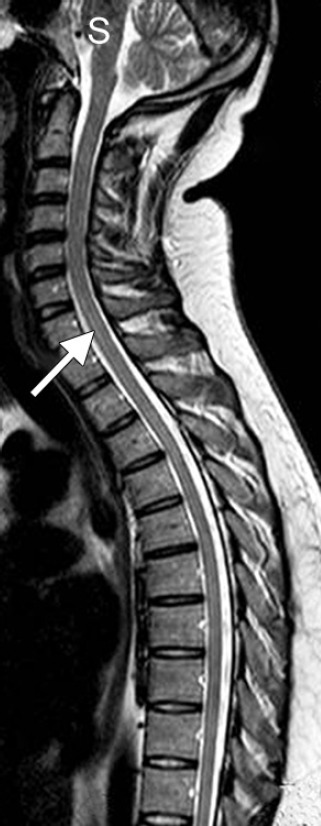
MRI brain and spine showing T1 hyperintensity in cervicodorsal area

A 28-year-old gentleman was admitted because of intermittent fever of 7 days duration and bilateral lower limb weakness of 1 day duration. Fever was low grade, maximum documented up to 101°F, and was associated with chills and headache. Lower limb weakness was symmetrical, proximal more than distal and was associated with tingling and numbness, band like sensation around the hips with bowel and bladder retention. Touch and pain sensations were decreased below level of L1. Deep tendon reflexes were 3+ in all four limbs and plantars were bilateral extensor. Laboratory investigations showed positive IgM serology for dengue. Cerebrospinal fluid (CSF) analysis showed 60 cells with 60% polymorphs and 40% lymphocytes; and normal protein and sugar level (Table 1). MRI brain and spine revealed T1 hyperintensity in cervico-dorsal region (Fig. 1). Serum antibody for neuromyelitis optica antibody was negative.

With a diagnosis of dengue-associated acute transverse myelitis (ATM), intravenous methylprednisolone 1 g daily was given for 5 days and patient had complete neurological recovery.

**Table 1:** Laboratory investigations

*Variable*	*Case 1*	*Case 2*
Hemoglobin (g/dL)	14.6	13.6
White cell count (per mm^3^)	4000	4700
Differential count (%)		
Neutrophils/lymphocytes	45/50	56/44
Platelet count (per mm^3^)	89000	37000
Aspartate aminotransferase (U/L)	154	355
Alanine aminotransferase (U/L)	67	194
CSF analysis		
Total cells	60	No cells
Differential counts (%)		
Neutrophils/lymphocytes	60/40	−
Protein/sugar (mg/dL)	40/54	27/11
Dengue serology for IgM	Positive	Positive
Dengue NS1 Ag	Negative	Positive

## CASE 2

A 48-year-old gentleman was admitted with complaints of fever for 5 days and weakness of all four limbs and neck for 1 day. Fever was intermittent, maximum recorded up to 102°F, and associated with chills. Patient had ascending weakness of all four limbs associated with weakness of neck muscles without any sensory, bladder or bowel involvement. There was no antecedent history of respiratory or gastrointestinal tract infection. Physical examination revealed areflexic flaccid paralysis of all four limbs (legs more than arms), neck muscle weakness and right lower motor neuron type facial nerve palsy. Laboratory investigations showed positive NS1 antigen and IgM serology for dengue. Cerebrospinal fluid was acellular with normal protein and sugar (Table 1). Nerve conduction study (NCS) showed pure motor axonal and demyelinating polyneuropathy. Patient was started on intravenous immunoglobulin as five daily infusions for a total dose of 2 g/kg body weight followed by a complete neurological improvement.

## DISCUSSION

Dengue is among the most common arboviral infections worldwide, and is endemic in many tropical regions. It is the prototypic arthropod-borne infection, notable for its marked heterogeneity of disease manifestations. Multiorgan involvement with capillary leak syndrome, internal organ bleeding, renal failure, liver injury, myocarditis, encephalopathy, and circulatory shock is characteristic of severe disease.

Dengue is a flavivirus and is closely related to other mosquito-borne neurotropic flavivirus causing significant neurological involvement such as Japanese encephalitis, Zika virus, West Nile virus, and St. Louis encephalitis virus.^1^

Most dengue infections are asymptomatic. The incidence of neurological manifestations ranges from 1 to 5% with encephalopathy or encephalitis being the commonest neurological manifestations.^1^ Parainfectious ATM and Guillain-Barré syndrome (GBS) are rare complications and found in less than 10% cases of dengue with neurological involvement.^2,3^ Both direct infection and post infectious immune mediated neural injury have been postulated as pathogenesis for these neurological complications.^1–3^

During Zika virus associated GBS outbreak, few cases were found to have dengue antibodies in serum or CSF, raising the possibility of primary dengue infection and false positive Zika virus serologies due to cross reactivity.^4^ Dengue-associated GBS cases were more common among adults than children. Nerve conduction study shows acute inflammatory demyelinating polyneuropathy in most cases. Majority of these cases have been treated with intravenous immunoglobulins. Plasmapheresis, corticosteroids or conservative treatment were used in some cases. Most of the cases have full recovery.^5^

Dengue associated ATM is diagnosed on the basis of spinal imaging and CSF analysis, and majority of these cases have been treated with intravenous corticosteroids with complete neurological recovery.^6^

Dengue virus infection may have wide spectrum of neurological manifestations apart from encephalopathy or encephalitis. Therefore patients with dengue infection, especially from endemic area, should be looked for early detection of these complications and for better understanding the impact of this disease.
